# Full Profiling of GE81112A, an Underexplored Tetrapeptide Antibiotic with Activity against Gram-Negative Pathogens

**DOI:** 10.1128/spectrum.02247-22

**Published:** 2023-05-04

**Authors:** Sören M. M. Schuler, Gerrit Jürjens, Alexander Marker, Ulrike Hemmann, Astrid Rey, Stéphane Yvon, Marjorie Lagrevol, Mohamed Hamiti, Fabian Nguyen, Rolf Hirsch, Christoph Pöverlein, Andreas Vilcinskas, Peter Hammann, Daniel N. Wilson, Michael Mourez, Sebastien Coyne, Armin Bauer

**Affiliations:** a Branch Bioresources of the Fraunhofer Institute for Molecular Biology and Applied Ecology, Giessen, Germany; b Sanofi-Aventis Deutschland GmbH, Frankfurt, Germany; c Sanofi R&D, Therapeutic Area Infectious Diseases, Marcy L’Etoile, France; d Gene Center, Department for Biochemistry and Center for Protein Science Munich, Ludwig-Maximilians-Universität München, Munich, Germany; e Institute for Insect Biotechnology, Justus-Liebig University of Giessen, Giessen, Germany; The University of North Carolina at Chapel Hill

**Keywords:** GE81112A, antibiotic, profiling

## Abstract

After the first total synthesis combined with structure revision, we performed thorough *in vitro* and *in vivo* profiling of the underexplored tetrapeptide GE81112A. From the determination of the biological activity spectrum and physicochemical and early absorption-distribution-metabolism-excretion-toxicity (eADMET) properties, as well as *in vivo* data regarding tolerability and pharmacokinetics (PK) in mice and efficacy in an Escherichia coli-induced septicemia model, we were able to identify the critical and limiting parameters of the original hit compound. Thus, the generated data will serve as the basis for further compound optimization programs and developability assessments to identify candidates for preclinical/clinical development derived from GE81112A as the lead structure.

**IMPORTANCE** The spread of antimicrobial resistance (AMR) is becoming a more and more important global threat to human health. With regard to current medical needs, penetration into the site of infection represents the major challenge in the treatment of infections caused by Gram-positive bacteria. Considering infections associated with Gram-negative bacteria, resistance is a major issue. Obviously, novel scaffolds for the design of new antibacterials in this arena are urgently needed to overcome this crisis. Such a novel potential lead structure is represented by the GE81112 compounds, which inhibit protein synthesis by interacting with the small 30S ribosomal subunit using a binding site distinct from that of other known ribosome-targeting antibiotics. Therefore, the tetrapeptide antibiotic GE81112A was chosen for further exploration as a potential lead for the development of antibiotics with a new mode of action against Gram-negative bacteria.

## INTRODUCTION

The spread of antimicrobial resistance (AMR) is becoming a more and more important global threat to human health. Since the “Golden Age” of antibiotic drug discovery in the 1940s to 1960s, no major novel class of antibiotics (except against tuberculosis) has been approved for Gram-positive bacteria for 40 years or for Gram-negative bacteria for 60 years, although the development of resistance to approved drugs is only a matter of time (1). New derivatives of already known classes displaying improved antibacterial spectra and pharmacokinetic (PK) properties represent the main progress since those times (1, 2). With regard to current medical needs, penetration into the site of infection represents the major challenge (3) in the treatment of infections caused by Gram-positive bacteria, while there are several compounds available, such as daptomycin, tigecycline, and linezolid, that still display good activities against, e.g., methicillin-resistant Staphylococcus aureus (MRSA) or vancomycin-resistant *Enterococcus* (VRE). Considering infections associated with Gram-negative bacteria, resistance is a major issue. Obviously, novel scaffolds for the design of new antibacterials in this arena are urgently needed to overcome this crisis (4). Such a novel potential lead structure is represented by the GE81112 compounds, which inhibit protein synthesis by interacting with the small 30S ribosomal subunit using a binding site distinct from that of other known ribosome-targeting antibiotics (5). Therefore, the tetrapeptide antibiotic GE81112A was chosen for further exploration as a potential lead for the development of antibiotics with a new mode of action (MoA) (6–8) against Gram-negative bacteria.

**FIG 1:**
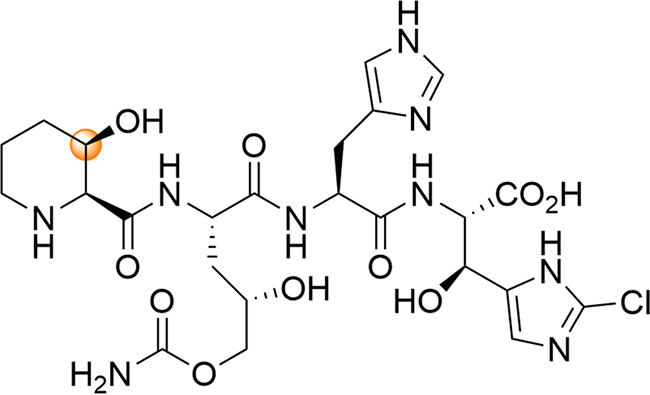
Revised structure of GE81112A (8). The highlighted C-atom has the inverse configuration, compared with that reported originally (6).

In 2006, GE81112 was isolated by Brandi et al. from a *Streptomyces* species as a mixture of three congeners (6). In our case, the target compounds were not available via fermentation in a sufficient amount for full lead profiling. Therefore, we selected GE81112A as the first target compound for total synthesis in order to perform extensive biological profiling. In addition to establishing the first synthetic route yielding 100 mg of GE81112A, we were able to revise the structure of the originally reported natural product (NP) by comparing nuclear magnetic resonance (NMR) analysis and activity data, as well by performing a translation inhibition assay (Fig. 1) (8).

Only a limited set of data on the PK and pharmacodynamic (PD) parameters of the GE family has been reported to date. In 2006, Brandi et al. introduced the compound class as protein translation inhibitors with a new MOA, inhibiting the formation of the prokaryotic 30S initiation complex, and with a binding site distinct from those of known antibiotics (6, 9). The cocrystal structure of the 30S ribosome with GE81112 has been also reported, giving further hints regarding the precise interactions of the compound with its target (10). When tested on bacterial cells, GE81112A showed selective activity against Gram-negative pathogens, with an important medium effect (6). While inactive against Escherichia coli in Mueller-Hinton II medium (MHII), the NP is active in minimal medium (MM) (6). The activity of the tetrapeptide in Dulbecco’s modified Eagle medium (DMEM) was reported by Maio et al. as a very similar finding in 2016 (11). That study also reported limited activity versus other E. coli strains and showed that the tetrapeptide was transported through the bacterial oligopeptide permease (Opp) (11). Investigating the inoculum effect, the authors observed the selection of spontaneous resistant mutants at a frequency of ~1 × 10^−6^. Characterizing the mutants as well as *opp*-deleted or -overexpressing strains, they confirmed the requirement for intact Opp for GE81112A uptake and inactivation of *opp* as a main mechanism of resistance (11). The aforementioned literature data represented the starting point of our in-house profiling to identify the key criteria, which had to be improved.

## RESULTS AND DISCUSSION

### *In vitro* activity.

For evaluation of the *in vitro* antibacterial activity of GE81112A, we selected 11 key pathogens from our standard Gram-negative testing panel to address two aspects, (i) confirming the reported activity of GE81112A in DMEM and inactivity in MHII and (ii) gaining a deeper insight into the spectrum of activity against enterobacteria, including carbapenem-resistant clinical isolates (Table 1).

**TABLE 1:** GE81112A MIC determinations with a panel of *Enterobacteriaceae*

Strain	Strain characteristic^*a*^	MIC (μg/mL)			
		GE81112A 1		Ciprofloxacin	
		MHII	DMEM	MHII	DMEM
E. coli MG1655		>64	1	≤0.016	≤0.016
E. coli ATCC BAA-2471	NDM-1	>64	4	>8	>8
E. coli DSM 46345		>64	>64	0.125	0.125
E. coli ATCC BAA-2469	NDM-1	>64	>64	>8	>8
K. pneumoniae ATCC BAA-2470	NDM-1	>64	>64	>8	>8
K. pneumoniae ATCC BAA-2342	KPC	>64	>64	>8	>8
E. cloacae ATCC BAA-2468	NDM-1	>64	>64	>8	>8
E. cloacae CIP 105879		>64	>64	≤0.016	0.032
P. mirabilis DSM4479		>64	64	≤0.016	0.032
C. freundii ATCC 8090		>64	32	≤0.016	≤0.016
P. stuartii ATCC 29851		>64	>64	8	8

a NDM, New Delhi metallo-β-lactamase 1; KPC, Klebsiella pneumoniae carbapenemase.

GE81112A was completely inactive in MHII against all strains tested. In DMEM, GE81112A was active only against E. coli MG1655 and NDM-1-producing E. coli ATCC BAA-2471. The lack of activity against other tested E. coli strains, as well as the MIC of 1 μg/mL in DMEM for E. coli MG1655, confirmed the findings of Maio et al. (11) No other enterobacteria were susceptible to GE81112. Summarizing our data, the activity spectrum of GE81112A is thus limited to only a subpopulation of E. coli isolates.

### Physicochemical properties.

As a very polar compound (log *D* at pH 7.4 = −1), GE81112A showed excellent solubility in both sodium chloride solution (0.9%) (>0.5 mg/mL) and phosphate buffer (pH 7.4) (>0.5 mg/mL). It is stable in phosphate buffer (pH 7.4), with 100% recovery after 24 h of incubation at 25°C, and in the presence of glutathione (GSH), showing no degradation or formation of adducts after incubation for 168 h with this nucleophile.

### *In vitro* early absorption-distribution-metabolism-excretion-toxicity testing.

The cell permeability of GE81112A was tested using a CaCo2/TC7 cell model (12). Absorption in the human intestine was predicted to be low, with an apparent permeability of 0.28 to 3.16 × 10^−7 ^cm/s using 1 or 20 μM GE81112A. However, this finding was not considered of relevance for further development because parenteral administration was targeted.

Metabolic stability was investigated using liver microsomes from human and animal species and human primary hepatocytes. Overall, oxidative metabolic lability was considered low at a concentration of 5 μM in all tested species, i.e., human, mouse, and rat liver microsomes.

At a concentration of 5 μM, the intrinsic clearance (CL_int_) using human primary plated hepatocytes was classified as low with 0.01 mL/(h × 1 million cells). Moreover, directly glucuronic acid-conjugated metabolites were not observed under our experimental conditions.

Competitive and time-dependent cytochrome P-450 (CYP) inhibition was investigated with human liver microsomes. Direct competitive and time-dependent inhibition of CYPA4 was not observed at concentrations of up to 50 μM.

GE81112A was also considered stable in blood plasma of human and animal species at a concentration of 1 μM at 37°C; 3% and 27% hydrolysis was observed after 1 and 4 h of incubation, respectively, in human blood plasma. No degradation was observed in mouse or rat blood plasma after 4 h.

**FIG 2:**
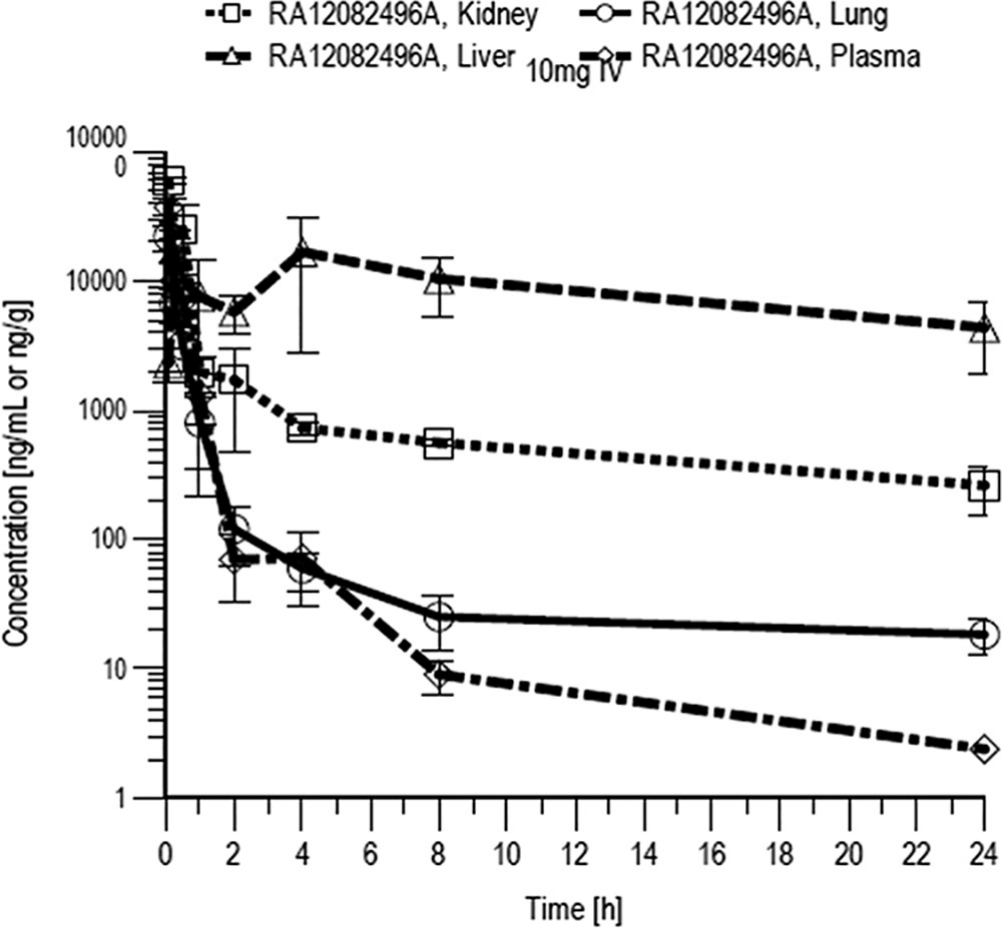
PK in mice after a single i.v. administration.

**FIG 3:**
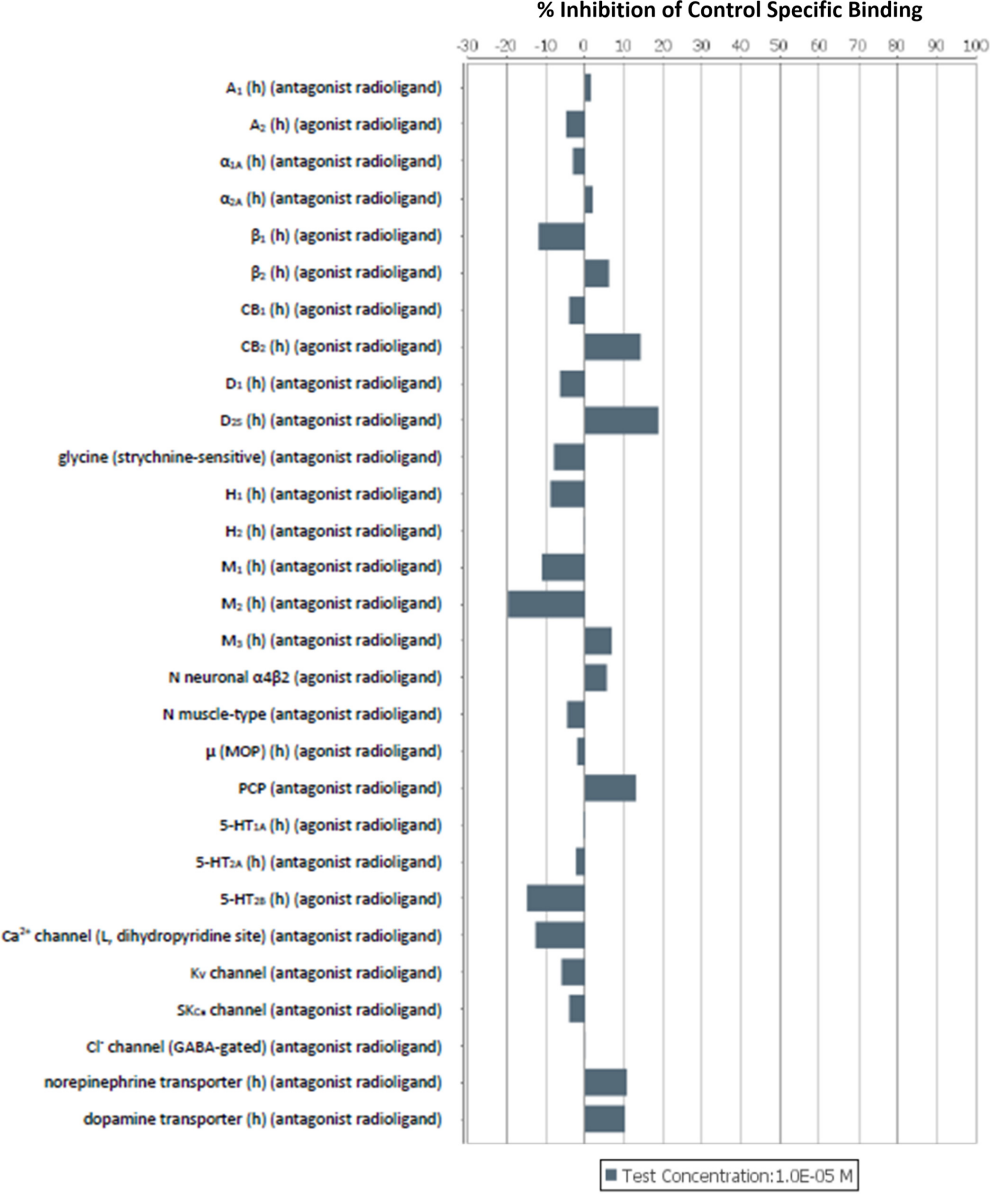
*In vitro* binding assays. Inhibitory effects of GE81112A were investigated for G-protein-coupled receptors for adenosine (A_1_ and A_2A_), androgens (α_1A_, α_2A_, β_1_, and β_2_), cannabinoids (CB_1_ and CB_2_), dopamine (D_1_ and D_2S_), histamine (H_1_ and H_2_), acetylcholine (M_1_, M_2_, and M_3_), opioids (μ-MOP), and serotonin (5-HT_1A_, 5-HT_2A_, and 5-HT_2B_), the ion channels Gly (rat), nAChR (α4β2), nAChR (muscle type), PCP (rat), Ca_v_1.2 (rat), K_v_ (rat), SK_Ca_ (rat), and GABA_Cl_ (rat), NET (GenBank accession number P23975), and DAT (GenBank accession number Q01959). The targets were human recombinant proteins unless a different species is stated. All tests were conducted at a peptide concentration of 0.01 mM according to CEREP procedures (https://www.eurofinsdiscovery.com/solution/safety-panels).

**FIG 4:**
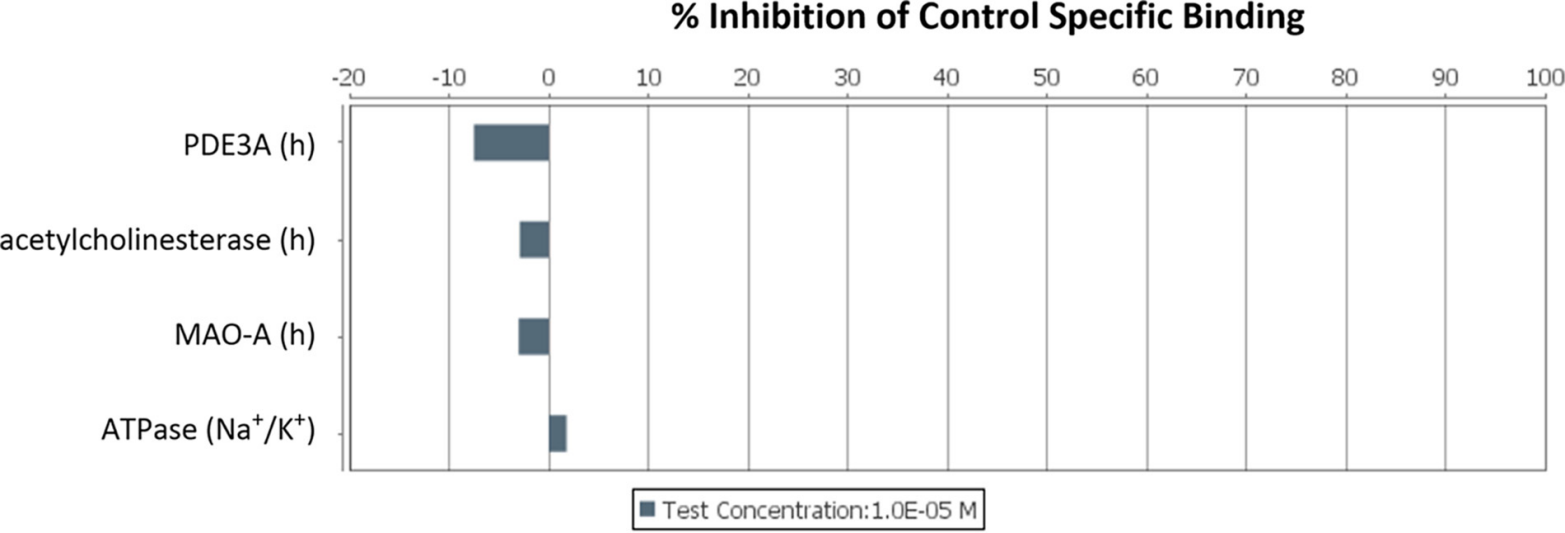
*In vitro* enzyme and uptake assays. Inhibitory effects of GE81112A were investigated for the enzymes PDE3A, acetylcholinesterase, MAO-A, and Na^+^/K^+^-ATPase (porcine). The targets were human recombinant proteins unless a different species is stated. All tests were conducted at a peptide concentration of 0.01 mM according to CEREP procedures.

The potential toxicity of GE81112A was assessed in primary rat hepatocytes and HepG2 cells. In both systems, a no observed effect concentration (NOEC) of 15.6 μM was determined. Considering this relatively low NOEC and the fact that PK studies in mice indicated accumulation of GE81112A in the liver (Fig. 2), additional studies with regard to liver uptake and toxicity would be required to assess the compound’s overall safety profile. Off-target inhibitory effects of GE81112A were also investigated via broad-spectrum *in vitro* pharmacological binding, enzyme, and uptake assays (Fig. 3 and 4). (The profiling was performed by CEREP, Celle-Le’vescault, France.) In all cases, no critical inhibiting effect was observed. Moreover, GE81112 did not inhibit selected hERG and other ion channels (hNa_V_1.5, hNa_V_1.2, hCa_V_3.1, and hK_V_4.3) at concentrations up to 30 μM. Furthermore, no mutagenicity was observed for GE81112A in the Ames II test in Salmonella enterica serovar Typhimurium. However, at concentrations of ≥0.75 μg/mL, a low bacterial background was noticed in the tests, likely due to the antibacterial activity of GE81112.

**FIG 5:**
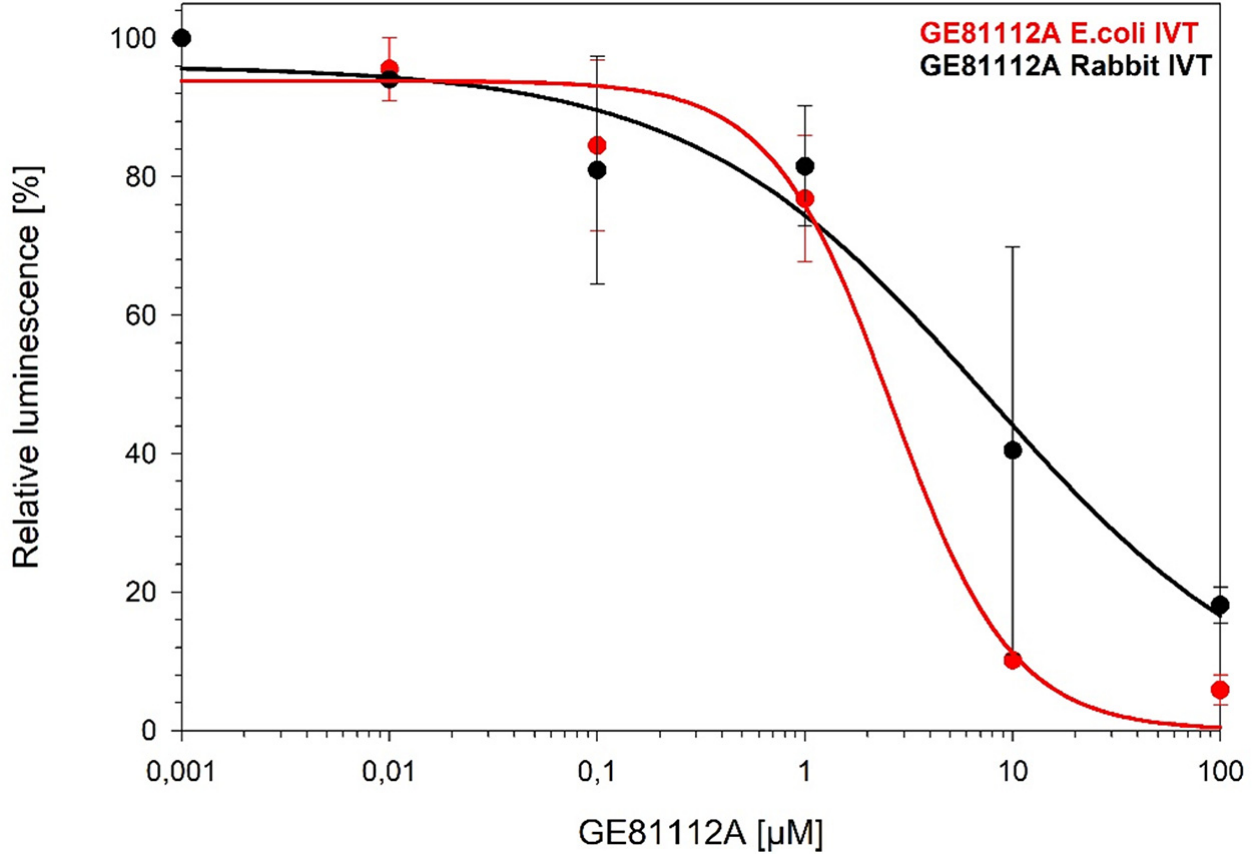
Inhibition by GE81112A using E. coli (red) and rabbit ribosomes (black), based *in vitro* translation (IVT) systems.

Considering that GE81112A is a protein synthesis inhibitor, we investigated the specificity of this inhibition by using lysate-based *in vitro* translation inhibition assays (8, 13, 14). Comparing bacterial systems with E. coli and mammalian systems using rabbit ribosomes, we observed comparable inhibition by GE81112A (Fig. 5). The NP cannot cross eukaryotic membranes due to its high polarity, and this has to be monitored carefully during compound optimization and development in order to avoid undesired side effects in follow-up derivatives.

### *In vivo* evaluation.

We first performed a tolerability study in uninfected mice by injecting GE81112A at up to 30 mg/kg, formulated in NaCl (0.9%), via intravenous (i.v.) bolus administration. At the highest tested single dose, mice did not show any sign of toxicity or adverse effects such as distress, behavioral signs of sickness, or weight loss.

In order to determine the PK parameters of GE81112A, a single i.v. dose of 10 mg/kg in solution was administered to male Swiss mice, and plasma, urine, and tissue concentrations were monitored over 24 h (Fig. 2). After i.v. dosing, the different parameters were investigated in more detail. A high distribution of GE81112A in the kidney points toward its excretion via urine. Therefore, urinary tract infections (UTIs) represent the most probable scope of application (Table 2).

**TABLE 2:** PK parameters after i.v. administration of 10 mg/kg GE81112A

Parameter	Finding
Plasma half-life (h)	4.5
Clearance (L/h/kg)	0.735
Volume of distribution (L/kg)	0.321
Proportion of dose recovered in urine (%)	30%
Tissue/plasma ratio	
Lung	0.515
Kidney	3.00
Liver	20.6

The overall plasma half-life was considered long, with a small volume of distribution. Tissue/plasma ratios were considered high with respect to liver tissue (tissue/plasma ratio of 20), suggesting an accumulation in liver. A tissue/plasma ratio of 2.9 was found in kidney tissue, suggesting urinary secretion; this was proved by showing that 30% of the administered dose was found in urine over the 24-h sample collection time.

**FIG 6:**
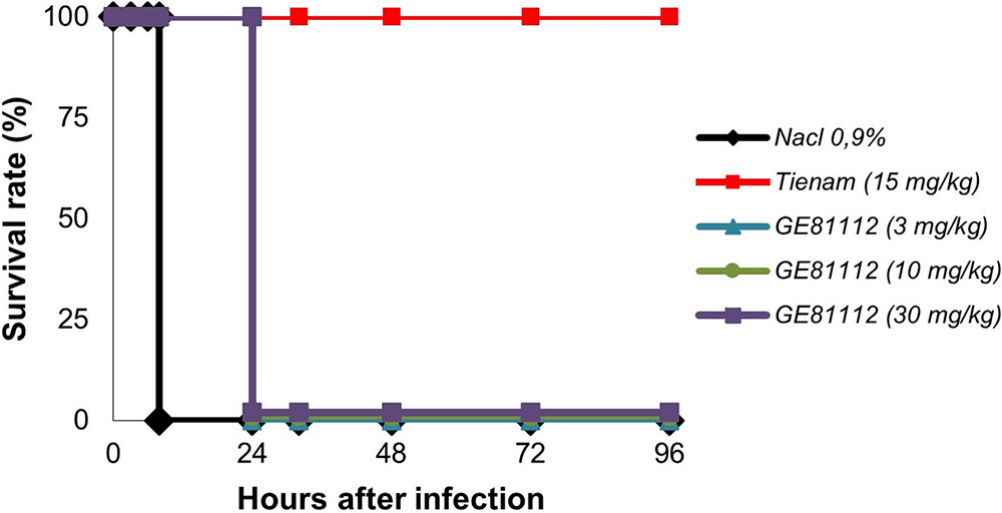
Dose-effect relationship of GE81112A for survival rates in the septicemia model.

**FIG 7:**
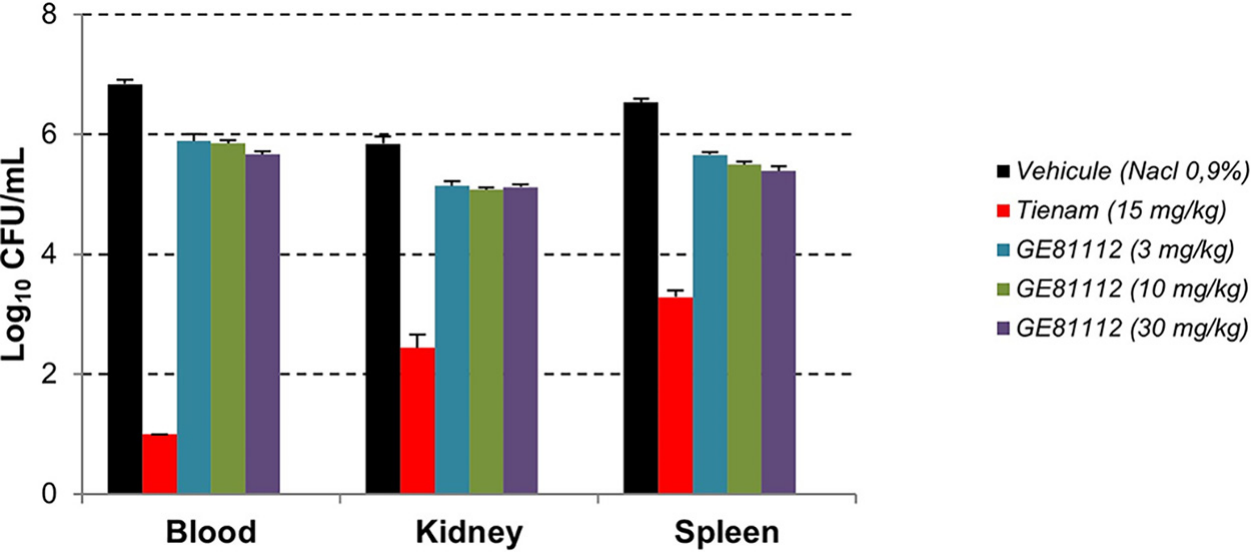
Dose-effect relationship of GE81112A for CFU loads in the septicemia model. The initial inoculum was 2 × 10^5^ CFU/mouse.

The efficacy of GE81112A was investigated in an E. coli ATCC 35218-induced mouse septicemia model. GE81112A was administered once by i.v. bolus at three different doses, 1 h postinfection. The bacterial burden was determined 4 h postinfection, and survival was monitored for up 96 h postinfection (Fig. 6 and 7).

While not impacting the survival of infected mice, GE81112 led to ~1-log_10_ CFU/mL reductions of bacterial burden in blood, kidney, and spleen, compared to vehicle alone, and kept the bacterial burden to the same level as the initial inoculum. This modest impact on bacterial burden appeared to be dose related.

### Summary.

The combination of (i) a reported high frequency of resistance linked to a unique nonessential transporter, (ii) a limited *in vitro* spectrum, (iii) high variability of activity among E. coli isolates, and (iv) weak *in vivo* efficacy impaired further straightforward development of GE81112A as an antibacterial lead compound. Our works, however, revealed disadvantages and critical issues regarding the parent hit compound GE81112A (Table 3), which have to be addressed in a follow-up project.

**TABLE 3:** Overview of investigated parameters

Profiling type and objective	Finding
*In vitro* activity	
Activity testing against 11 enterobacterial species	− (limited spectrum)
Physicochemical properties	
Log *D*	+
Solubility (NaCl and phosphate buffer)	+
Stability (phosphate buffer and GSH)	+
*In vitro* eADMET^*a*^	
CaCo_2_ permeability	− (not relevant)
Metabolic stability	+
CYP inhibition	+
Human liver microsomes	+
Plasma stability and hemolysis	+
Translation inhibition	+/− (to be monitored)
Cytotoxicity (rat hepatocytes/HepG2 cells)	+/− (to be monitored)
Pharmacological off-target profiling	+
Off-target ion channel inhibition	+
Mutagenesis (Ames II test)	+
*In vitro* assays	
Tolerability	+
PK study	+/− (administration)
E. coli-induced septicemia model	− (weak effect)

a eADMET, early absorption-distribution-metabolism-excretion-toxicity.

In particular, improving bacterial penetration by attaining independence from the unique transporter could further increase the activity in complex culture media and decrease the risk of resistance development. Considering the activity of GE81112A on both bacterial and mammalian ribosomes, specificity toward bacterial cells should also be warranted in order to limit cytotoxicity. Maintaining a PK profile compatible with the development of a treatment for complicated UTIs (cUTIs) (high kidney levels, urinary excretion after i.v. administration, and high polarity of the compound) would be another interesting property to look for. Based on this full profiling and especially the good physicochemical profile, we conclude that, even though the compound has some major issues, mainly related to its uptake mechanism and excretion, it will be an interesting candidate as a starting point for development if these problems can be addressed in a medicinal chemistry campaign, considering the scarcity of novel compounds active against Gram-negative organisms. Uptake via another transporter therefore will play a key role in the future development of this molecule regarding its larger potential, and it is currently being investigated by a group with one of the authors.

Therefore, based on the full profiling of GE81112A, as well as the previously published total synthesis, which enables access to larger amounts of compound and provides a basis for derivatization, the tetrapeptide GE81112A series could represent a promising starting point for chemical modifications and optimization programs to develop a new-MoA antibiotic targeting Gram-negative organisms.

## MATERIALS AND METHODS

### MIC determination.

MICs were determined by adapting the protocol from CLSI guidelines (Table 1; 15). Briefly, from an overnight preculture, 49.2 μL of an inoculum of 5 × 10^5^ CFU/mL in MH broth or DMEM was dispensed into 384-well plates, and 0.8 μL of compounds solubilized in dimethyl sulfoxide (DMSO) and serially diluted was added to the bacterial cultures. The plates were incubated at 37°C for 16 to 18 h. MICs were determined by measurement of the optical density at 600 nm (OD_600_). The experiments were performed in duplicate. Commercially available strains were obtained from the American Type Culture Collection (ATCC), the German Collection of Microorganisms and Cell Cultures (DSMZ), and the Biological Resource Center of Institut Pasteur (CRBIP). E. coli MG1655, E. coli ATCC BAA-2471, E. coli DSM 46345, E. coli ATCC BAA-2469, Klebsiella pneumoniae ATCC BAA-2470, K. pneumoniae ATCC BAA-2342, Enterobacter cloacae ATCC BAA-2468, E. cloacae CIP 105879, Proteus mirabilis DSM 4479, Citrobacter freundii ATCC 8090, and Providencia stuartii ATCC 29851 were selected as screening strains, and ciprofloxacin was utilized as the positive control. The MIC values for ciprofloxacin were consistent with reported values (data not shown) and thereby validated the experiment performed. No shift of activity between MHII and DMEM was observed for the selected positive control.

### Determination of log *D* at pH 7.4.

Log *D* values at pH 7.4 were determined with a standardized high-performance liquid chromatography (HPLC) method, essentially as described by Krass et al. (16) The calculation of the log *D* values for measured compounds is performed by comparison of the retention times with standard compounds of known distribution coefficients for distribution between 1-octanol and water at pH 7.4. The highly polar GE81112A displays a log *D* value below −1.

### Solubility tests.

**(i) Solubility in phosphate buffer (pH 7.4).** For calibration, approximately 2 mg of GE81112A was weighed into a HPLC glass vial. DMSO was added to reach a target concentration of 2 mg/mL. Dilutions for calibration (10 μg/mL, 50 μg/mL, and 100 μg/mL) were prepared by adding adjusted volumes of DMSO and were correlated with ultraperformance liquid chromatography (UPLC)-UV peak areas at 220 nm. For solubility determinations, 2 mg of GE81112A was weighed into a HPLC glass vial. Phosphate buffer (pH 7.4) was added to reach a target concentration of 2 mg/mL. The vial was shaken for 16 h at 25°C, with protection from light, on an Eppendorf thermomixer at 600 rpm, followed by centrifugation at 10,000 rpm for 5 min at 25°C and filtration through a 0.45-μm filter. The filtrated supernatant was analyzed (UPLC-UV, λ = 220 nm), and the concentration of the analyte in the filtrate was calculated based on the peak area/real concentration ratio from previous calibration runs.

**(ii) Solubility in 0.9% NaCl solution.** To obtain a crystal-like form of the compound, GE81112A was dissolved in water followed by freeze-drying. Afterwards, 510 μg GE81112A was transferred to a glass vial with a microinsert, and 50 μL NaCl (0.9%) was added.

### Stability at pH 7.4 for 24 h.

Five microliters of a GE81112A stock solution (10 mM in DMSO) was added to 195 μL of phosphate buffer (pH 7.4) in a V-shaped multititer plate (96 wells). The plate was stored for 24 h at 25°C, with protection from light, after which the aged solution was analyzed by UPLC-UV (λ = 220 nm). The calculation of the remaining compound was performed based on the UV peak area ratios before and after incubation.

### Chemical stability in the presence of GSH.

Based on a stock solution of 0.2 mg GE81112A in 1,548 μL methanol, three different samples were prepared in triplets (test mixture: 100 μL stock solution, 2 μL DMSO, and 1 μL DMSO containing 1.5 GSH equivalents; reference sample A: 200 μL stock solution; reference sample B: 200 μL stock solution and 2 μL DMSO). All samples were measured at three different time points (0 h, 24 h, and 168 h) on a MaXis II system (Bruker).

### Cellular permeability assay using the CaCo2/TC7 cell line.

CaCo2/TC7 cells were seeded in a Falcon 24-well multiwell insert system with 1.0-μm-pore high-density polyethylene terephthalate (PET) membrane plates, at a density of 60,000 cells per well in DMEM (high glucose, with Glutamax I, without HEPES, with 20% fetal calf serum [FCS], and with penicillin-streptomycin). The cells were incubated for at least 21 days to ensure complete differentiation, polarization, and formation of a tight barrier layer. Permeability assays were performed in Hanks’ balanced salt solution (HBSS) with sodium taurocholate (186 μM), HEPES (10 mM), and bovine serum albumin (BSA) (0.5% [wt/vol]), adjusted to pH 7.4 with sodium hydroxide (1 N). GE81112A at 1 and 20 μM was assayed for 120 min in the apical-to-basolateral direction with an apical pH of 7.4 and a basolateral pH of 7.4, without a BSA gradient (0.5%) on either side. After the incubation period, apical and basolateral concentrations of GE81112A were determined using liquid chromatography-tandem mass spectrometry (LC-MS/MS). The mean apparent permeability coefficient (*P*_app_) (single point, in centimeters per second) was calculated as *P*_app_ = *R*_2_/([*D*_0_] × *S* × *t*), where *R*_2_ is the receiver quantity of compound after 2 h (peak area or calculated concentration) and [*D*_0_] is the donor concentration of test solution (peak area or calculated concentration).

### Metabolic stability.

Microsomal preparations from human and animal species were used at a protein concentration of 1 mg/mL, and the cofactor NADPH was added to a final concentration of 1 mM to assay for CYP and flavin-containing monooxygenase (FMO) lability. GE81112A at 5 μM was incubated for 20 min at 37°C. The enzymatic reaction was initiated by a rapid temperature increase in a water bath at 37°C. It was stopped by a rapid temperature decrease in an ice bath at 0°C and subsequent addition of 5 volumes of precooled acetonitrile (500 μL). Loss of parent GE81112A concentration, as a percentage, was determined using LC-MS/MS measurements, where 100% was the measurement at time zero.

For hepatic *in vitro* CL_int_ studies, primary cultures of cryopreserved human or animal species hepatocytes were seeded at a density of 200,000 cells per well and cultured overnight in collagen-coated 48-well plates in modified Ham’s F-12/William’s E medium. Disappearance kinetics of 5 μM GE81112A were sampled over a 24-h period. Dextromethorphan, midazolam, phenacetin, and tolbutamide (5 μM each) were used as reference compounds. Acetonitrile was used to terminate the incubation, and samples were centrifuged at 2,000 × *g* for 10 min after the addition of an appropriate internal standard. Supernatants were separated by LC using an AERIS peptide XB-C_18_ column (3.6 μm, 50 by 2.1 mm; Phenomenex, Aschaffenburg, Germany) with the following gradient of buffer A (water) and buffer B (acetonitrile): 0 to 5 min, 95% buffer A; 20 to 25 min, 5% buffer A. Both solvents were supplemented with 0.1% formic acid. The remaining parent peptide was quantified by LC-MS/MS using a Q Exactive hybrid quadrupole-Orbitrap device (Thermo Fisher Scientific). *In vitro* hepatic CL_int_ was calculated as dose/area under the concentration-time curve from 0 to 24 h (AUC_0–24_), where dose is the concentration of GE81112A determined in the time zero sample and classified using reference compounds for low, moderate, or high CL_int_.

### CYP inhibition IC_50_ shift assay.

To examine the potential for time-dependent and direct competitive CYP3A4 inhibition, the reaction mixture was prepared with a human liver microsome concentration of 0.1 mg/mL. GE81112A was added to final concentrations of 50, 25, 10, 5, 2, 0.4, and 0.08 μM. Mixtures were brought to 37°C, with the addition of an NADPH-generating system (EDTA, 1 mM; MgCl_2_, 3 mM; β-NADPH, 1 mM; glucose 6-phosphate, 5 mM final concentration) in water to one set of reaction mixtures and water to a second set of reaction mixtures. The reaction mixtures were preincubated for 45 min. A CYP isoform-selective substrate (3 μM midazolam or 50 μM testosterone) was added to the reaction mixtures, and NADPH was added to the second set of reaction mixtures for an additional 10-min incubation period. The reactions were stopped by the addition of 200 μL internal standard solution in acetonitrile.

Both incubation plates (for midazolam and testosterone) were centrifuged at 3,000 rpm for 10 min. The samples were analyzed using LC-MS/MS. Parallel analysis of the CYP3A4 substrate midazolam and the CYP3A4 substrate testosterone was performed. The calculations of 50% inhibitory concentration (IC_50_) values and IC_50_ shift values were performed by using 4-paratemeter curve fitting.

### Plasma stability.

For plasma clearance, peptides were dissolved as 1 mM stock solutions in sterile deionized water and incubated at 37°C in 500 μL plasma derived from different species (human, mouse, and rat) at a final concentration of 5 μM. At different time points (0, 1, and 4 h), 100-μL samples were transferred to 500 μL ethanol containing 0.5% (vol/vol) ammonia, and plasma proteins were precipitated for 20 min at 1,735 × *g*. Each 10-μL supernatant sample was separated on a Triart C_18_ column (1.9 μm, 20 by 2 mm; YMC, Kyoto, Japan) with an ascending acetonitrile gradient in water (supplemented with 0.1% formic acid), at a flow rate of 500 μL/min. Samples were analyzed in triplicate by LC-MS/MS as above, and the stability of the parent peptides was evaluated based on the comparison of blank samples with incubated samples.

### Hemolysis of human erythrocytes.

The hemolytic activity of GE81112A was tested in a 96-well round-bottom microtiter plate, in a final volume of 100 μL. Erythrocytes were isolated from fresh citrate-stabilized blood from human donors by repeated centrifugation (5 min at 500 × *g*) and washing with phosphate-buffered saline (PBS). To obtain the final suspension, the isolated erythrocytes were diluted 1:50 in PBS. GE81112A was dissolved in sterile water, and we prepared a 3-fold 1:2 dilution series in the concentration range of 2,048 to 256 μg/mL, in a volume of 50 μL. We then added 50 μL of the erythrocyte suspension to each well, and the lidded test plates were incubated for 5 h at 37°C in 85% relative humidity, with shaking at 180 rpm. The erythrocytes were then pelleted, and 80 μL of the supernatant was transferred to a new 96-well microtiter plate to quantify the released hemoglobin by turbidity measurement at 540 nm. The percent hemolysis caused by GE81112A was calculated relative to the values of the blank and the positive control (Triton X-100).

### Translation inhibition assay.

The bacterial and mammalian lysate-based *in vitro* translation reactions were performed using the RTS 500 E. coli high-yield (HY) kit (catalog number BR1400201; biotechrabbit) and the rabbit reticulocyte lysate system (catalog number L4960; Promega), respectively, as described previously (13). Briefly, 6-μL reaction mixtures with increasing concentrations of GE81112A were mixed according to the manufacturer’s instructions and incubated for 1 h at 30°C, with shaking at 750 rpm, to express the reporter protein firefly luciferase. A total of 0.5 μL of each reaction mixture was stopped with 7.5 μL kanamycin (50 μg/μL). All samples were diluted with 40 μL of luciferase assay substrate (catalog number E1500; Promega) in a white 96-well, chimney-style, flat-bottom microtiter plate (catalog number 655095; Greiner). The luminescence was immediately measured using a Tecan Infinite M1000 plate reader. Relative values were determined by defining the luminescence value of the sample without inhibitor as 100%.

### Cytotoxicity for HepG2 cells and primary rat hepatocytes.

The toxicity of GE81112A toward human hepatocellular carcinoma HepG2 HB-8065 (ATCC) cells and freshly isolated primary Sprague-Dawley rat hepatocytes (Charles River) was assessed by using the CellTiter-Glo ATP-monitoring kit (Promega) and by quantifying the ability to store the dye neutral red (neutral red uptake [NRU] solution; Sigma-Aldrich, St. Louis, MO, USA). The assay was conducted in 96-well microtiter plates in a test volume of 200 μL. GE81112A was tested in an 8-fold 1:2 dilution series with a final concentration range of 400 to 1.56 μM. HepG2 cells or primary hepatocytes were maintained at 37°C in 5% CO_2_ in DMEM-F-12 medium containing 1% nonessential amino acids, 1% sodium pyruvate, and 10% heat-inactivated FCS. Prior to each test, 100 μL of culture medium was added to each well (each containing about 20,000 cells), and the plates were incubated for 16 h as described above. GE81112A was diluted in culture medium to obtain appropriate concentrations and was added to the wells as six replicates. Ketoconazole was used as a positive control for toxicity, and PBS was used as the blank. After incubation for 24 h as described above, cell viability was calculated either by cell lysis and subsequent luminometric quantification of the ATP concentration in each sample or by measurement of the amount of neutral red taken up by the cells. NRU was measured at 540 nm (Tecan Genios Pro) after 3 h of incubation with NRU solution and subsequent cell lysis. The stated NOEC values refer to the highest sample concentration with cell viability of >80%.

### Pharmacological off-target profiling.

Pharmacological off-target profiling was conducted by CEREP (Celle-Le’vescault, France) to investigate inhibitory effects against the targets specified in Fig. 3 and 4. All tests were conducted at a concentration of 10 μM (42.0 and 43.3 mg/L for LS-sarcotoxin and LS-stomoxyn, respectively). We selected 19 G-protein-coupled receptors (acetylcholine, adenosine, androgens, cannabinoids, dopamine, histamine, opioids, and serotonin), eight ion channels (Gly, nicotinic acetylcholine receptor [nAChR], phencyclidine [PCP], Ca_v_1.2, K_v_, SK_Ca_, and γ-aminobutyric acid-gated chloride channels [GABA_Cl_]), two transporters (dopamine transporter [DAT] and noradrenaline transporter [NET]), and four enzymes (phosphodiesterase [PDE], acetylcholinesterase, monoamine oxidase (MAO), and Na^+^/K^+^-ATPase) for testing.

### Off-target ion channel profiling.

The effect of GE81112A on the hERG and other ion channels (hNaV1.5, hNaV1.2, hCaV3.1, or hKV4.3) was investigated using an automated patch-clamp method, as described by Houtmann et al. (17). GE81112A was diluted in a 5-fold 1:3 dilution series with a final concentration range of 30 to 0.12 μM in extracellular medium (150 mM NaCl, 4 mM KCl, 2 mM CaCl_2_, 1 mM MgCl_2_, 10 mM HEPES, 10 mM glucose, 0.06% Pluronic F-68, 0.3% residual DMSO). The hERG channel was constitutively expressed in Chinese hamster ovary (CHO) cells (CHO hERG Duo; B’SYS GmbH, Witterswil, Switzerland). CHO cells were grown at a concentration of 8 × 10^6^ CHO cells/mL in QPlates (Sophion/Biolin Scientific, Ballerup, Denmark) in Ex-Cell animal component-free CHO medium (Sigma-Aldrich) supplemented with 25 mM HEPES, 100 U/mL penicillin-streptomycin, and 0.004% soybean trypsin inhibitor. To each well, we added extracellular medium containing the desired concentration of GE81112A. The peptide-hERG interaction was quantified by recording the tail current following repolarization of the hERG channels using a QPatch HTX station (Sophion/Biolin Scientific). The IC_50_ values were determined using the values from three replicates of the GE81112A concentration series with respect to the terfenadine citrate positive control and extracellular medium (blank).

### Ames II test with Salmonella enterica serovar Typhimurium.

The test was carried out with and without metabolic activation by S9 liver homogenate from rats that had been pretreated with Arcolor, S9 mix. The Salmonella enterica serovar Typhimurium tester strains included the mixed strains TA7001 to TA7006 for detection of base pair substitutions and TA98 for detection of frameshift mutations (18). Bacterial cultures (from frozen stocks) were grown overnight and tested for genotype conformation. Test compound diluted in DMSO was tested at 16, 50, 160, 500, 1,600, and 5,000 μg/mL with each strain, both in the presence and in the absence of the exogenous hepatic microsomal fraction (S9) from Aroclor 1254-treated rats (obtained as described above). A liquid preincubation protocol was employed, in which the bacteria, test chemical, and S9 mixture or sodium phosphate buffer (pH 7.4) were added for culture in a 24-well plate and incubated at 37°C ± 2°C for 90 min in an environmental incubator with shaking at 100 rpm. Then, 2.8 mL of a pH indicator solution (Merck KGaA, Darmstadt, Germany) (bromocresol purple) containing trace amounts of histidine and biotin (ready-to-use commercial solution) was added to each well, and all samples were then transferred into a 384-well plate. Plates were incubated at 37°C ± 2°C for 48 h. Toxicity was assessed by evaluating the density of growth of the background lawn of bacteria. Mutagenicity was assessed by counting the number of wells with color changes (purple or yellow) or the number of easily visible colonies (revertants).

### *In vivo* tolerability studies.

All animal studies were performed in accordance with relevant guidelines and regulations. A 30 mg/kg dose of GE81112A, formulated in 0.9% NaCl, was administered to noninfected Swiss mice (*n* = 3), using a single i.v. bolus. Adverse events and animal weights were monitored for 24 h after administration.

### Ethical statement.

In vivo studies were performed in an AAALAC international accredited animal facility and approved by the Sanofi Pasteur Ethics Committee for Animal Experimentation in compliance with the European Directive 2010/63/EU as published in the French Official Journal of February 7th, 2013. All efforts were made to reduce the use of animals and minimize pain and distress in application of the 3Rs principles.

### PK analysis.

Plasma, urine, and tissue concentrations and PK parameters for GE81112 were assessed after a single i.v. administration of a 10-mg/kg solution to male Swiss mice. Briefly, 5 mL/kg of a 2-mg/mL solution of GE81112A was administered i.v. to fed male Swiss mice (weight of 20 g, obtained from Janvier; *n* = 24 mice). Samples were collected at 0.08, 0.25, 0.5, 1, 2, 4, 8, and 24 h postadministration for bioanalysis. Bioanalytical samples (plasma, urine, and tissue) were analyzed for the concentration of GE81112 using an exploratory LC-MS/MS method, and PK parameters were estimated using the PK software Phoenix WinNonlin v6.4, using a noncompartmental model and a linear trapezoidal interpolation calculation. The studies were ethically approved by the German governmental authority (protocol number FH/2002; Regierungspräsidium Darmstadt).

### E. coli-induced septicemia model.

Male Swiss mice, 6 to 8 weeks of age (5 per group), were infected through intraperitoneal injection of 0.2 mL E. coli ATCC 35218 at 2 × 10^5^ CFU/mouse in 5% mucin. GE81112A was given i.v. at 3, 10, and 30 mg/kg/day, at 1 and 3 h postinfection. As a control, imipenem was given i.v. at 15 mg/kg/day. Mice were monitored for body conditions and weight. Groups of animals were euthanized 4 h postinfection in order to determine the bacterial burden in blood, kidney, and spleen. Other groups of mice were monitored for survival until 96 h postinfection.
